# Soil water content effects on net ecosystem CO_2_ exchange and actual evapotranspiration in a Mediterranean semiarid savanna of Central Chile

**DOI:** 10.1038/s41598-018-26934-z

**Published:** 2018-06-05

**Authors:** Francisco J. Meza, Carlo Montes, Felipe Bravo-Martínez, Penélope Serrano-Ortiz, Andrew S. Kowalski

**Affiliations:** 1 Facultad de Agronomía e Ingeniería Forestal, Pontificia Universidad Católica de Chile, Santiago, Chile; 2 Centro Interdisciplinario de Cambio Global, Pontificia Universidad Católica de Chile, Santiago, Chile; 3 NASA Goddard Institute for Space Studies, New York City, NY USA; 4 Departamento de Ecología, Universidad de Granada, Granada, Spain; 5 Andalusian Institute for Earth System Research (CEAMA-IISTA), Granada, Spain; 6 Departamento de Física Aplicada, Universidad de Granada, Granada, Spain

## Abstract

Biosphere-atmosphere water and carbon fluxes depend on ecosystem structure, and their magnitudes and seasonal behavior are driven by environmental and biological factors. We studied the seasonal behavior of net ecosystem CO_2_ exchange (NEE), Gross Primary Productivity (GPP), Ecosystem Respiration (RE), and actual evapotranspiration (ETa) obtained by eddy covariance measurements during two years in a Mediterranean Acacia savanna ecosystem (*Acacia caven*) in Central Chile. The annual carbon balance was −53 g C m^−2^ in 2011 and −111 g C m^−2^ in 2012, showing that the ecosystem acts as a net sink of CO_2_, notwithstanding water limitations on photosynthesis observed in this particularly dry period. Total annual ETa was of 128 mm in 2011 and 139 mm in 2012. Both NEE and ETa exhibited strong seasonality with peak values recorded in the winter season (July to September), as a result of ecosystem phenology, soil water content and rainfall occurrence. Consequently, the maximum carbon assimilation rate occurred in wintertime. Results show that soil water content is a major driver of GPP and RE, defining their seasonal patterns and the annual carbon assimilation capacity of the ecosystem, and also modulating the effect that solar radiation and air temperature have on NEE components at shorter time scales.

## Introduction

Arid and semiarid regions of the world cover more than 40% of the total land surface, are characterized by low precipitation amounts and high evaporation rates, are exposed to high hydroclimatic variability and usually exhibit relatively low soil fertility, factors that condition biomass accumulation. These regions are of particular interest for the global carbon budget as it has been estimated that their ecosystems are responsible for up to 20% of terrestrial net primary productivity^1^.

Recent studies suggest that current global trends in the carbon sink of the biosphere are dominated by semiarid ecosystems^2^. In these regions, NEE is controlled by precipitation, and droughts are identified as major elements that reduce GPP by limiting photosynthetic rates, and shortening the length of the growing season^3,4^. This is the case of the exceptionally positive anomaly in global carbon uptake registered in 2011, which is mainly ascribed to increased productivity of semiarid vegetation in the Southern Hemisphere, and especially to the effect of wet conditions in Australia during a La Niña year^5,6^. However, since La Niña years are typically associated with negative rainfall anomalies in regions such as Central Chile, this major carbon uptake cannot be generalized. Precipitation is a commonly used climatic variable to explain the behavior of xeric vegetation. However, plant growth and development directly depend on soil water content (SWC) and plant water uptake. Therefore, rainfall records can be misleading as intense precipitation events do not always result in a proportional increase of SWC either because of low infiltration rates (e.g. because of soil compaction) or a relatively small soil water holding capacity. Additionally, in areas with strong rainfall seasonality, the occurrence of precipitation is not always associated with the onset of the vegetation growth, exhibiting lags of several weeks and even months, and thus reducing the explanatory power of precipitation records.

In Mediterranean environments, the dry season is associated with warm temperature that often result in strong water stress conditions for plants due to the high atmospheric evaporative demand and restricted soil water content^7^. As a direct consequence of climate change, it is expected that Mediterranean-type regions will face a systematic decrease in rainfall amounts^8^ and a warming-enhanced evapotranspiration^9^, with a resulting decrease in soil water content that will affect carbon uptake^10,11^. Furthermore, other studies^12^ indicate that aridity overrides the climatological sensitivity of NEE to temperature in dry areas. This could be the case of semiarid Chile, where increasing dryness in spring and summer has been reported during recent decades^13^, which are consistent with climate change projections^14^, suggesting high vulnerability of NEE as climate becomes drier and warmer^15^.

Multiple studies have assessed NEE patterns in Mediterranean ecosystems of Europe^16–18^ and California^7,19^, but Mediterranean ecosystems in Latin America such as those found in Central Chile remain poorly studied. Despite similarities in environmental conditions and vegetation traits, fluctuations in total amount and timing of winter/spring precipitation, the literature points to significant among-site differences in annual NEE, mainly due to fluctuations in winter/spring precipitation, both in terms of total amount and timing^20,21^. Ecosystems respond directly to shortages in water availability by reducing their photosynthetic capacity and respiration, and by showing carry-over effects associated with disturbance in stored soil moisture, organic matter and nutrients, which can lead to an increase in plant mortality^22^. In the same way, reduction of annual carbon uptake by up to 45% has been quantified in Mediterranean cork oak woodlands as a result of a lower photosynthetic rate and leaf area duration^11,23^. The same effect was observed on woody species^3^, together with a change from net carbon sink into net source in a grassland, likely due to lower soil exploration by roots. Similarly, a shift to net carbon source was reported in a grassland in California due to a shorter growing season^24^.

**Figure 1:**
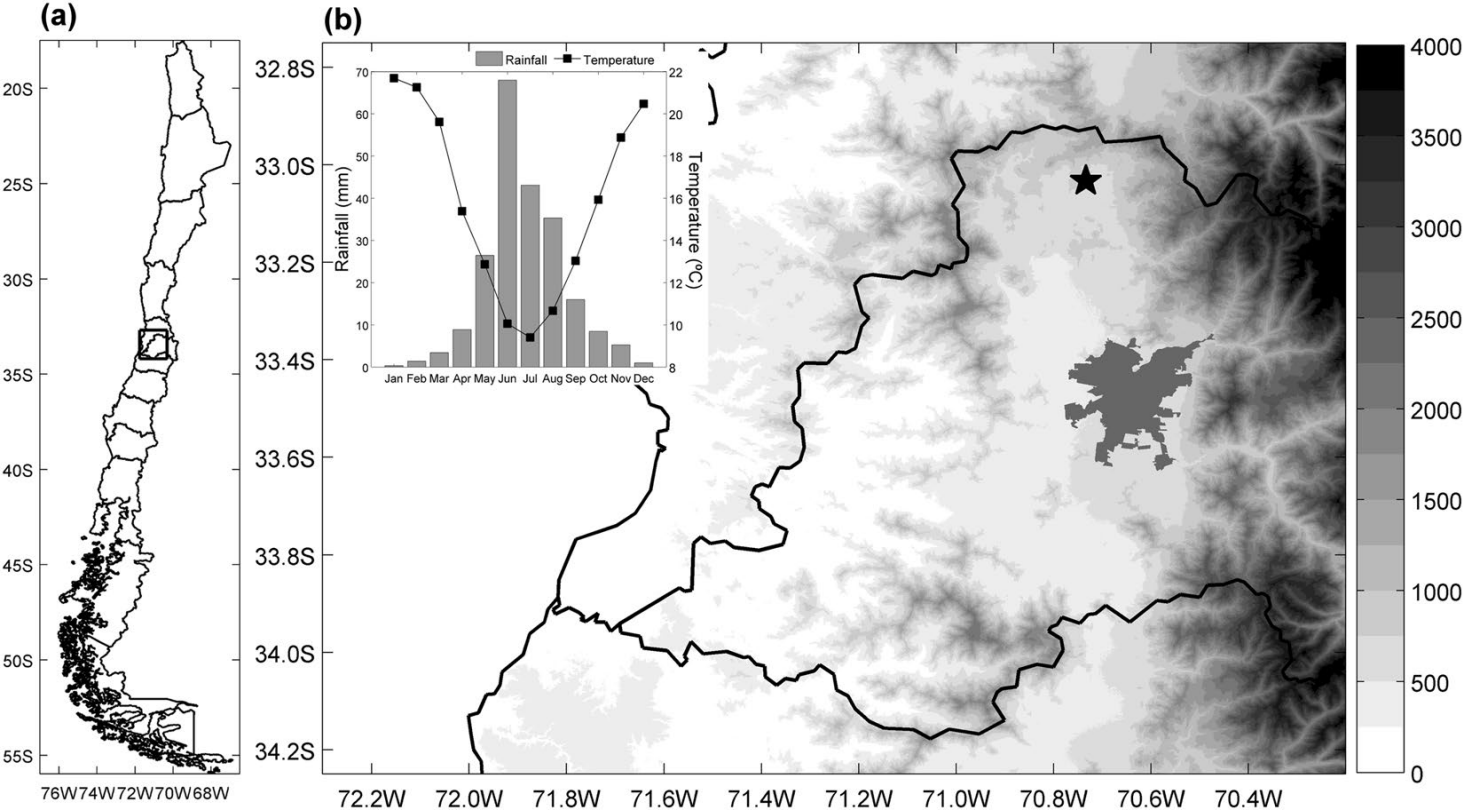
(**a**) Location and (**b**) topographic map (bar in m.a.s.l.) of the study area in Central Chile, including the monthly climograph showing the mean annual cycle of rainfall and air temperature (1994–2012). The *star* in (**b**) shows the measurements site, the thick lines are administrative divisions and the darker polygon in (**b**) shows the city of Santiago. We used Advanced Spaceborne Thermal Emission and Reflection Radiometer (ASTER) Global Digital Elevation Model Version 2 (GDEM V2) data retrieved from the online Global Data Explorer, courtesy of the NASA Land Processes Distributed Active Archive Center (LP DAAC), USGS/Earth Resources Observation and Science (EROS) Center, Sioux Falls, South Dakota, https://gdex.cr.usgs.gov/gdex/.

Given the relevance of carbon uptake in dry ecosystems, the strong seasonality of precipitation, and the increasing occurrence of drought spells in Central Chile, our objective was to assess the seasonal variation of NEE and its components (GPP, RE) as well as ETa in a Mediterranean *Acacia caven* savanna and their relations to soil water content. The study area is characterized by strong seasonality with more than 85% of total precipitation concentrated in winter, a fact that was particularly evident during the observation period where only few rains fell in autumn. Our underlying hypothesis is that soil water content is an important driver of NEE during winter periods, modifying the response of GPP and RE to other environmental factors such as photosynthetically active radiation (PAR) and temperature. As a consequence, the highest photosynthetic activity of this ecosystem occurs between July and August. This study uses eddy covariance time series of CO_2_ and water vapor obtained from a Mediterranean *Acacia caven* savanna (Fig. 1) during two consecutive hydrological years.

## Results

### Environmental conditions during the experiment

**Figure 2:**
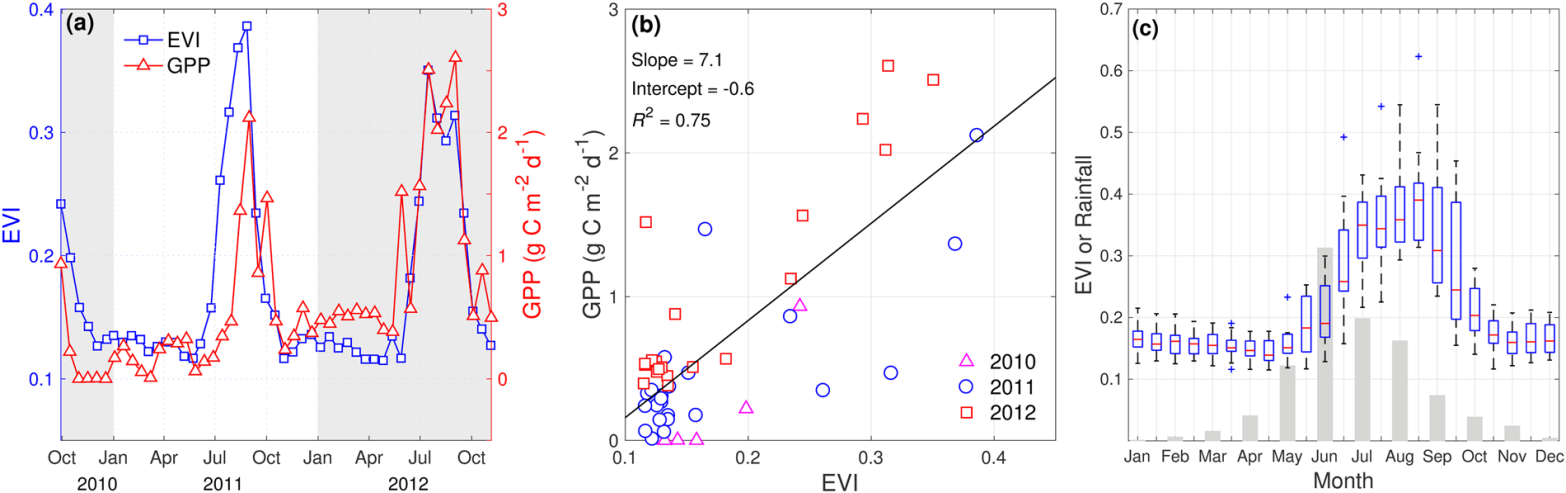
(**a**) Time series of daily values of GPP and EVI for the study site (**b**) Relationship between EVI and GPP values and (**c**) Boxplots of annual distribution of the Enhanced Vegetation Index (EVI), gray bars represent the relative distribution of annual precipitation during the year (average monthly values divided by total sum of precipitation). Central mark shows the median and the edges are the 25th and 75th percentiles; dashed lines extend to the most extreme values not considered outliers, and outliers are plotted individually (plus sign).

The Enhanced Vegetation Index (EVI) was used as a proxy to relate patterns of ecosystem fluxes to the vegetation cycle. EVI and GPP showed similar temporal variability (Fig. 2a EVI peaked close to 0.4 in August-September). The linear relationship between EVI and GPP (Fig. 2b) allow us to detect ecosystem photosynthetic activity especially when herbaceous species are present, accounting for 75% of GPP variance.

The annual cycle of monthly mean EVI values (Fig. 2c) shows strong seasonal variability, closely associated with the seasonal precipitation distribution. Minimum EVI values occur in autumn (April, 0.15 ± 0.01) and maximum values in late winter (August, 0.40 ± 0.08), before and after the rainy season, respectively. We observe that the highest photosynthetic activity of the system occurs between late winter and early spring (July, August and September), while the rest of the year is characterized by steady and small NEE values that are interrupted only by short pulses of rain triggering peaks in ecosystem respiration.

**Figure 3:**
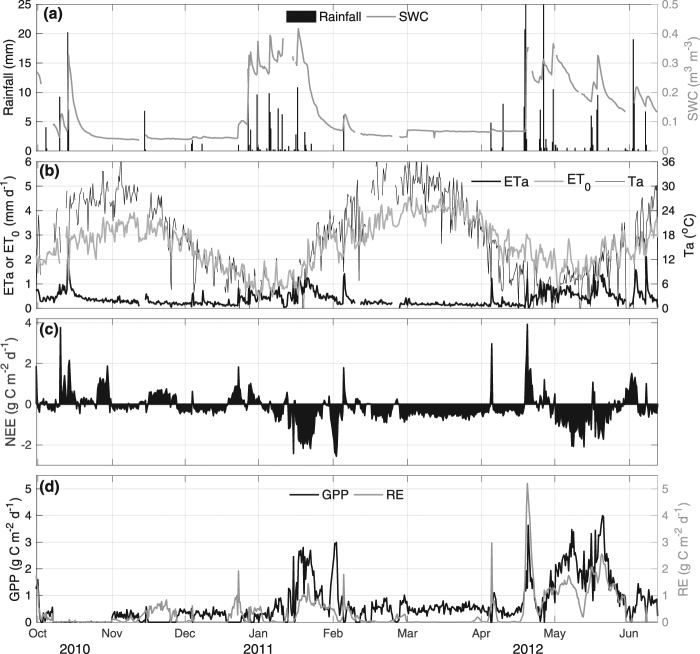
Time series of daily values of (**a**) rainfall and volumetric soil water content (SWC), (**b**) actual (ETa) and reference (ET_0_) evapotranspiration, and air temperature (**c**) net ecosystem exchange (NEE), (**d**) gross primary productivity (GPP) and ecosystem respiration (RE).

The seasonal course in daily rainfall and soil water content (SWC) (Fig. 3a) shows typical Mediterranean-type features, with most events occurring during winter and early spring (from June/July to Aug/Sep.), coinciding with minimum values of daily mean air (*T*_*a*_ Fig. 3b) and soil temperature (*T*_*s*_, data not shown) of around 5 °C. Although SWC responds rapidly to precipitation (Fig. 3a), some rainfall events are of such intensity that maximum soil water holding capacity is rapidly reached and excess water is lost due to runoff. The dry season extends from summer to early autumn, and is characterized by very few and short rain events (Fig. 3a), high average *T*_*a*_ and *T*_*s*_ (up to 25 °C and 37 °C, respectively), and vapor pressure deficit (VPD) (up to 2–2.5 kPa). For clear-sky days, maximum PAR and average net radiation (*R*_*n*_) values in summer were near 2000 µmol m^−2^ s^−1^ and 160 W m^−2^, respectively, versus 1000 µmol m^−2^ s^−1^ and 25 W m^−2^ in winter.

### Seasonal patterns of CO_2_, water vapor fluxes and main drivers

Daily ETa (Fig. 3b) showed an increase from the end of the warm season to a maximum in winter and early spring (15-day moving average of 0.1 mm d^−1^ and 1 mm d^−1^, respectively) when SWC was between 0.2–0.3 m^3^ m^−3^. Conversely, reference evapotranspiration (ET_0_ which corresponds to the atmospheric water demand acting on a short grass actively growing that is regarded as a reference vegetation surface) reaches its maximum during the warm season as a response to available energy and atmospheric demand. This pattern is only interrupted by soil water recharge after rainfall events.

The annual course of NEE (Fig. 3c) shows that the *Acacia* ecosystem varies from a net sink to a carbon source depending on the time of year, with a lower/higher magnitude during the warm/cold season. Greater fluctuations are observed from 2010 through 2011, while a steadier carbon sink behavior remained throughout 2012. Between summer and autumn (January to June), when *Acacia* almost exclusively composes vegetation, NEE rates were similar (varying from −0.7 to 0.7 g C m^−2^ d^−1^). In 2011, fluctuations were mainly associated with sporadic rain events (Fig. 3a). However, the effect of rainfalls on NEE depends on the season: in autumn and winter, rainfalls were associated with an increase in carbon uptake, while in the warm/dry season they generated net carbon losses.

A maximum carbon assimilation rate (-NEE) of around 2.5 g C m^−2^ d^−1^ was observed during winter and early spring (July to September). As shown in Fig. 3c, the first rain events in autumn/winter were followed by positive values of NEE, showing a similar response of GPP and RE to increasing SWC as in precedent months, likely as a response to temperatures allowing microbial respiratory activity to exceed photosynthesis (Fig. 3d).

**Figure 4:**
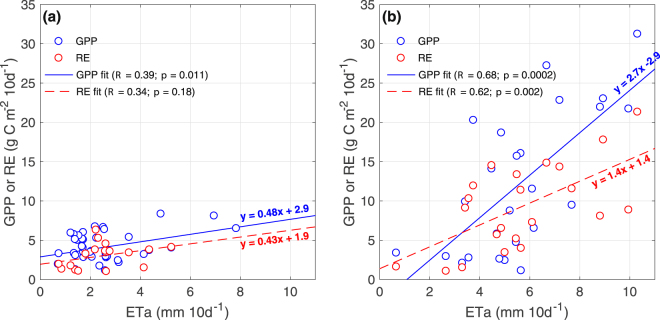
Relationship between 10-day accumulated ETa and carbon fluxes (GPP, RE) during (**a**) the dry and (**b**) the wet period of the year. *R* and *p* denote the Pearson’s correlation coefficient and *p*-value of statistical significance, respectively.

Following previous studies^25^, the effect of SWC on NEE was assessed by analyzing the functional relationship between ETa, GPP and RE. This was applied to 10-day accumulated values, separately for the dry (October-May) and wet (June–September) season. A moderate slope and a positive y-axis intercept for both GPP and RE characterizes the relationship between ET and carbon fluxes during the dry period (Fig. 4a). On the other hand, a higher slope was obtained between ETa and carbon fluxes during the wet period (Fig. 4b), and, unlike the dry period, a negative intercept was found for the ETa:GPP relationship.

**Figure 5:**
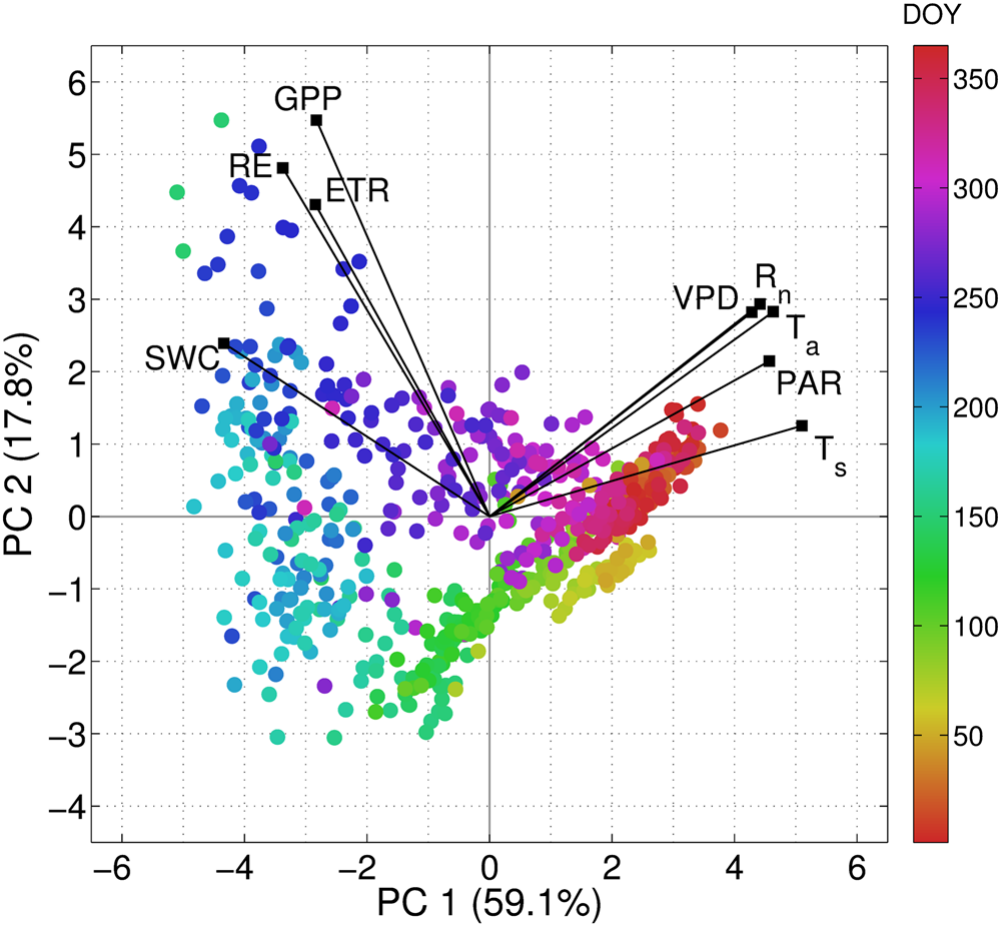
Biplot displaying the two first principal components (PC 1 and PC 2) and their corresponding explained variance (in %) derived from a PCA applied to daily gross primary productivity (GPP), ecosystem respiration (RE), actual evapotranspiration (ETa), air temperature (*T*_*a*_), soil temperature (*T*_*s*_), net radiation (*R*_*n*_), photosynthetically active radiation (PAR), soil water content (SWC) and vapor pressure deficit (VPD) (*n* = 683). Vectors denote the contribution of a single variable to the PC. Color bar denotes the day of the year (DOY) for data pairs.

To further explore how NEE components and environmental variables are related on a seasonal basis, a Principal Component Analysis (PCA) was applied to centered and variance-scaled daily GPP, RE, ETa, *T*_*a*_, *T*_*s*_, *R*_*n*_, PAR, SWC and VPD. Results are displayed in Fig. 5 for the two first principal components (PC; eigenvalues >1, 76.9% of total variance) and for the remaining PC in Table S1. The biplot shows that PC 1 separates carbon fluxes, ETa and SWC from *T*_*a*_, *T*_*s*_, *R*_*n*_, PAR and VPD, which in turn are poorly correlated (angle between vectors near 90°). On the other hand, the direct relationship between SWC and carbon and water vapor fluxes at the actual time scale and analyzed period is supported by their high correlation (angle close to zero). In addition, the sequential ordering according to the day of the year (DOY) of data pairs accounts for strong seasonality, where dry/hot and wet/cold periods are clearly differentiated. Although explaining less variance (17.3%), PC 2 describes variations in GPP, RE and ETa, given their higher correlation with this component (Table S1).

**Figure 6:**
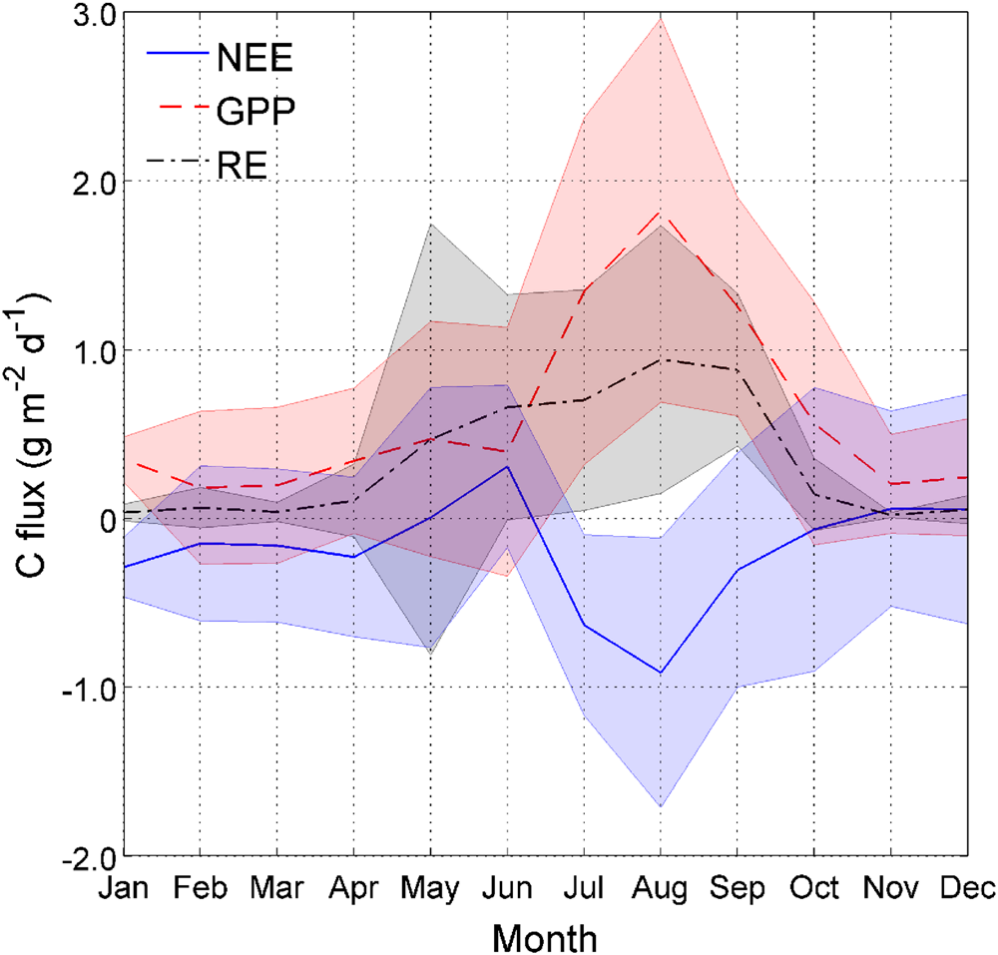
Annual cycle for monthly-averaged daily values of NEE, GPP and RE. Shaded intervals represent the standard deviation.

The above results are summarized as the average monthly annual cycle of NEE, GPP and RE (Fig. 6). NEE and its components show little variability from January to April, after which the opposing relationship between GPP and RE generates a maximum of NEE in June following the onset of the rainy season. The average maximum assimilation period during winter and early spring (June–September) begins after the divergence between GPP and RE rates to reach their maxima in August, to then remain close to zero in summer.

The effect of SWC on RE was further evaluated looking at the specific period of winter early spring that presents the maximum NEE magnitude (July–September). Table 1 shows the parameters of the Van’t Hoff model fitted to the observed nighttime RE values using linear regression. We used bin widths of 2 K and a minimum bin size of n = 10 to calculate mean values of RE and to reduce undesired effects of heteroscedasticity.

**Table 1:** Parameters of the Van’t Hoff model representing the influence of temperature on RE for different soil water content categories for the period from June to September.

RWC class	R_eco,ref_ (μmol m^−2^ s^−1^)	B	R^2^	RMSE (μmol m^−2^ s^−1^)
Low	0.832	0.028	0.46	0.123
Medium	1.073	0.081	0.88	0.156
High	1.340	0.100	0.87	0.163

In winter (July to September) SWC has an effect on ecosystem respiration (RE), increasing its value at the reference temperature (R_eco, ref_ ; T_ref_ = 283.16 K), and almost doubling it when relative soil water content is above 0.6 in comparison to the lower end. In addition, the rate at which temperature modifies ecosystem respiration (parameter B) increases as SWC increases, this effect is particularly evident when changing from low to medium relative soil water content (RWC, equation 2) values. Because of the strong seasonality in rainfall and SWC, most of the observations in the other periods (spring, summer and autumn) fall within a RWC class of low being difficult to assess the effects of soil water content on RE, only autumn data allow us to compare the parameters of the Van’t Hoff model between RWC classes low (R_eco,ref_ = 0.38 and B = −0.004) and medium (R_eco,ref_ = 0.52 and B = 0.032).

**Figure 7:**
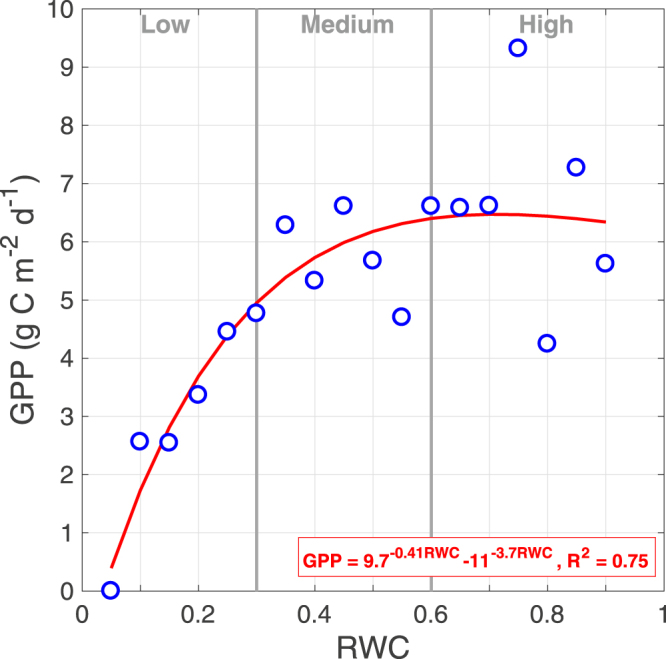
Relationship between instantaneous GPP (expressed as mass of carbon per unit area per day) and Relative Soil Water content (RWC).

The parameters of Table 1 were then used to calculate RE during daytime and combined with NEE records to calculate GPP. We plotted GPP values against RWC (Fig. 7), with values grouped in 0.05 (dimensionless) increments to achieve a relatively homogeneous bin size.

The relationship between GPP as a function of RWC indicates that soil moisture has a great effect on the ability of the ecosystem to capture carbon.

## Discussion

Integrating over the year, we found annual NEE values of −53 g C m^−2^ y^−1^ and −111 g C m^−2^ y^−1^ and ETa values of 128 and 139 mm for 2011 and 2012, respectively. The study was carried out during a severe drought (2010–2012). In this period, annual rainfall was found to be below normal: 9% deficit in 2010 (223 mm), 49% deficit in 2011 (111 mm), and 36% deficit in 2012 (155 mm), the years represent the initial period of a severe drought that extends to 2013 and beyond^26^, comparable only to that registered between 1967 and 1969, where three consecutive years showed significant deficits. This feature suggests that the *Acacia* ecosystem acts as a net carbon sink, with large seasonal variability. Since eddy covariance (EC) measurements were taken during a particularly dry period, greater carbon uptake should be expected for close-to-normal or wet years, as suggested by the linear relationship between GPP and ETa that in turn responds to precipitation and SWC. Notwithstanding this, the annual carbon fluxes are within the range observed for similar water-limited Mediterranean ecosystems. For instance, decreases in uptake from 140 g C m^−2^ y^−1^ to 28 g C m^−2^ y^−1^ (2003–2006) and from 388 g C m^−2^ y^−1^ to 214 g C m^−2^ y^−1^ (2011–2012) were found for a similar Mediterranean oak woodland in Portugal due to the effect of drought^3,11^.

A noticeable feature in measured carbon fluxes is the high seasonality of their annual course. Although this can be immediately related to the annual cycle in precipitation and the associated suppression of soil and plant metabolism during the warm season, it can be also explained by the phenological lag between the herbaceous cover and the *Acacia* activity. Thus, the first rains in autumn give way to germination and subsequent growth of grasses until the beginning of the dry season when *Acacia* begins its growth^27^. This seasonal behavior generates an increase in biomass that is coupled to the rainy season in winter, which has been also described for similar Mediterranean ecosystems^28^. As a consequence, annual carbon flux variations are dominated by rainfall during winter and early spring months (which maintain relatively high SWC levels), when maximum rates of NEE, GPP, RE and ETa occur, due to the activity of the well-adapted understory developing under unshaded and nitrogen-rich conditions prior to the leafing-out of *Acacia*^29^. In addition, the net increase in late winter productivity by the herbaceous stratum might be enhanced by the increasing soil water retention capacity and nutrient content due to the decomposition of recently shed *Acacia* leaves during the period of higher water availability^27,30^.

Steady rates of carbon assimilation were observed during the dry season when *Acacia* photosynthesis is dominant and SWC restricts soil and plant respiration while grasses are senescent. This dry-season sink behavior coupled with high atmospheric evaporative demand might be possible thanks to deep soil exploration by the root system of *Acacia*. In addition, the absence of precipitation in summer suggests a possible water supply to *Acacia* trees originating from sources such as groundwater and its redistribution by roots^31,32^, which would explain persistent photosynthetic rates during the dry season. However, further research is needed to validate this hypothesis. Furthermore, a moderate slope characterizes the relationship between ET and carbon fluxes during the dry period (Fig. 4a), indicating a small change in GPP/RE as water availability increases, and suggesting a weak dependence of the seasonal ecosystem functionality on sporadic rains. This can also be inferred from the positive y-axis intercept for both GPP and RE in Fig. 4a, indicating photosynthetic and respiratory capacity when ETa is close to zero. In addition, the slightly higher slope and y-axis intercept of ETa:GPP than ETa:RE would account for a weak and almost invariant carbon sink capacity during this period. It is worth mentioning that although the relationship between ETa and RE is not statistically significant (*p* = 0.18), there is a trend that should be addressed in future studies that examine, for example, a larger data sample.

During the onset of winter rainfalls both GPP and RE experienced substantial increases as a result of SWC increase, contributing to the net carbon uptake and sink capacity in spite of the lower temperature and available energy for photosynthesis, as reported in the literature^33^. Comparing the 2011 and 2012 years, both GPP and RE are higher in the cold season of 2012 which had more rainfall.

As shown in Fig. 4b, the existence of an ETa value below which RE exceeds GPP could be related to the existence of a threshold ETa and therefore SWC necessary for photosynthesis activation after dry periods or for seed germination in the transition from dry/warm to wet/cold season, whose magnitude (ETa ~1 mm 10 d^−1^) might be related to direct soil and/or canopy evaporation when GPP approaches zero. This is relevant considering the actual and projected aridity over the region and the associated probability of a reduced carbon uptake capacity or even conversion to a carbon source^10^, represented by the difference between the two lines in Fig. 4b, as precipitation decreases in upcoming decades. The latter represents a major issue also because of the possible southward expansion of the Mediterranean-type climate^34^ and the invasive nature of *Acacia* sp^35^.Furthermore, the threshold in available water (the intersection between the lines in Fig. 4b) could be reached later at the beginning of the wet season in a drier climate, shortening the period of higher net carbon assimilation. The slope obtained between ETa and carbon fluxes during the wet period suggests a greater sensitivity to water availability when changes in stomatal conductance or biomass can drive abrupt changes in carbon and water fluxes, as observed at the annual scale^21^. In turn, the steep slope for the case of ETa:GPP accounts for the dependence of carbon absorption capacity on water available to the ecosystem. Unlike the dry period, a negative intercept in the relationship ETa:GPP indicates that there is a value of ETa below which little or no growth can be observed. Similar to the dry season, the y-axis intercept in ETa:RE would account for a net carbon loss when ETa approaches zero. Moreover, considering the intercept between the two lines as the point from which GPP is higher than RE, an ETa of 2.5 mm 10 d^−1^ can be estimated as a threshold above which the ecosystem becomes a carbon sink, whose magnitude depends on available water.

The PCA applied to carbon and water fluxes and their environmental drivers (Fig. 5, Table S1) suggests a seasonally varying relationship, with SWC as the main factor controlling the seasonal behavior of carbon and water vapor fluxes. As such, the great importance of soil water content as a factor controlling RE has been reported in dry lands^36^, when warm-season rain events activate autotrophic and heterotrophic respiration causing short RE pulses and carbon release of around 1–1.5 g C m^−2^ d^−1^, whose magnitude depends on rainfall amounts^17^.According to the literature^37^, carbon emissions associated with short precipitation pulses are important in arid regions with sparse vegetation given the low water infiltration of bare soil versus below-canopy soil. In addition, authors^38^ estimated at 59 mm the necessary spring rainfall to produce a net CO_2_ uptake in a semiarid shrub in Arizona, and that short rainfall pulses triggered 40% of CO_2_ emissions from grassland in southeastern Spain for the period 2009–2013^39^. Moreover, RE pulses were described as resulting in a change from a CO_2_ sink to a net source for a steppe ecosystem^40^ and in a Mediterranean grassland^41^. Also, soil moisture was reported as the main driving factor of growing season NEE in a sagebrush-steppe ecosystem, and that VPD is important when the ecosystem is not limited by SWC^42^. In this sense, the high evaporative demand and dryness in summer prevent the development of the herbaceous stratum, which could compensate this carbon loss, so that this effect should be limited to the duration of surface soil wetness from field capacity to a minimum value^43^ (i.e., wilting point). Regarding the warm season in 2012, no precipitation events were observed, so that the steady negative NEE accounts for the moderate CO_2_ sink capacity of the ecosystem despite the seasonal dryness and high VPD, which is in agreement with the well-known adaptations to *Acacia* in terms of deep rooting and soil exploration, while herbaceous species are senescent^44^.

The relationship between SWC and RE/GPP suggests an important modulation of the effect that temperature and PAR can have on those variables respectively (and therefore on NEE) which might compromise the sink capacity of this system under future climate scenarios. Climate change projections from general circulation models for the region show higher temperatures and decreasing rainfall amounts for this region^45^, which in turn create a scenario where soil water content diminishes (as effective precipitation is reduced and temperature causes higher evaporation rates). Moreover, even for the period of greater activity we can expect that both RE and GPP will be reduced, creating a scenario where NEE changes could substantially compromise the sink behavior of this system.

## Conclusions

Eddy covariance CO_2_ fluxes were measured in a Mediterranean *Acacia caven* savanna ecosystem in Central Chile to examine their seasonal patterns and main drivers during 2011 and 2012. Results showed annual carbon uptake of the *Acacia* savanna of 53 g C m^−2^ in 2011 and 111 g C m^−2^ in 2012 with strong variability due mainly to differences in precipitation and therefore in SWC. Although carbon and water fluxes correlate with environmental variables during the year, the annual NEE pattern was strongly influenced by SWC and the ecosystem phenology.

Results from this study provide an overview about the seasonal behavior of carbon exchange by the *Acacia* savanna ecosystem of Central Chile during years of water limitation and in face of climate change. Greater sensitivity to SWC was observed in GPP and RE during the wet/cold season when grasses are present, versus the slow, steady dry/warm season, which nonetheless strongly determines the carbon sink capacity. This is in direct relation with winter precipitations that determine the herbaceous growth as the SWC determines its development. The interannual carbon balance indicates that the sink capacity of the *Acacia* savanna is mainly driven by its productivity during winter, which is thereby relevant from a climate change perspective considering the projected decrease in precipitation over the region. Finally, longer measurements of CO_2_ fluxes are needed in order to improve knowledge about the Mediterranean-type ecosystems of Central Chile and to better understand the sink/source cycles of the *Acacia* savanna under contrasting precipitation regimes (e.g. El Niño/La Niña years).

## Materials and Methods

### Study site description

Measurements were conducted on a 24-ha Mediterranean savanna located at the foot of the Andes in Central Chile (33°02′S 70°44′W, elevation 660 m.a.s.l., terrain slope 3%; Fig. 1). In this region, the destruction and replacement of the native evergreen sclerophyllous forests by croplands and animal husbandry have given way to an early successional plant community, locally known as *espinal*, which covers about 2 million ha of the Mediterranean perarid areas near 30°S (100–150 mm of annual rainfall) to perhumid zones near 36°S (700–1200 mm of annual rainfall)^27,44^. This ecosystem represents the most widespread agroforestry system of Central Chile, coexisting with herbaceous and sparse woody plants, all exposed to grazing pressure^29,46^.

Vegetation is composed of both woody and herbaceous species dominated by the thorny leguminous tree *Acacia caven* (Mol.) (Fabaceae; hereafter *Acacia*). *Acacia* is a woody plant of about 3 m height forming a sparse-open canopy (30% crown cover) arboreal stratum^29^. As a leguminous plant, it is a highly efficient nitrogen-fixing shrub whose semi-deciduous foliage allows frequent nitrogen recycling from lower to upper soil horizons^27^. During the rainy season (austral winter), this phenomenon promotes the development of an herbaceous stratum composed by annual C3/C4 herbs and grasses including *Anoda sp*, *Erodium moschatum*, *Trifolium sp*, *Oxalis sp*, *Urtica urens* and *Helenium aromaticum*.Other woody plants (<10% of cover) include *Porlieria chilensis*, *Prosopis chilensis* or *Proustia cuneifolia*^44^.

The climate of the study region is semiarid Mediterranean-type, with monthly average temperature ranging from 9.4 °C to 21.7 °C (15.8 °C annual mean). Precipitation shows an average value of 234 mm (1994–2012; data presented below) and is highly concentrated in the cold season. Interannual variations are largely regulated by the El Niño-Southern Oscillation, which induces positive/negative precipitation anomalies during the El Niño/La Niña phases. The soil of the site is classified as a shallow Vertic Calcixerolls (Mollisol), with low organic matter content (2.9%), clay loam texture (30:32:38 sand:silt:clay) and a C:N ratio of 12.

### Data collection

#### Eddy covariance measurements and data processing

Turbulent CO_2_ and energy fluxes (latent and sensible heat) were measured from October-2010 to October-2012 by an eddy covariance (EC) tower installed 6 m above the soil surface on the *Acacia* site, consisting of a CSAT3 (Campbell Scientific; Logan, UT, USA; hereafter CSI) sonic anemometer and an Li-7500(Li-Cor, Lincoln, NE, USA) open-path infrared gas analyzer (IRGA). Data were sampled at 10 Hz and stored half-hourly as average net fluxes. Data post-processing indicated that the site has a 700-m fetch, and footprint analyses showed that fluxes originated exclusively from the *espinal*. The Flux-Source Area model^47^ allowed verifying that for periods of relative stability (friction velocity >0.1 m s^−1^ and <0.2 m s^−1^, negative sensible heat flux), when measured fluxes are generated farthest from the tower, they originated from within the fetch (data not shown). For such stable conditions, a maximum distance to 80% source area isopleths of 300 m was estimated.

CO_2_ and energy fluxes data were corrected for density perturbations and coordinate rotation^48,49^ and checked for quality-control^50^.Data generated by dirty lenses were discarded after a filter based on IRGA diagnostics^51^, and averaging periods with insufficient turbulence (friction velocity <0.1 m s^−1^) were rejected^52^. Gaps in the database (~20%) were mainly due to dirty lenses (53%), power failures (28%), anomalous peaks due to morning flush^53^ and weak turbulence (19%). An energy balance closure of 68% was obtained, which falls within the range reported in the literature^54^.

CO_2_ and water vapor balances were calculated after gap filling. The gap-filling method delivers not only an estimate of missing values but also its standard error (σ_i_). To estimate the uncertainty in annual values, we assumed independence in the errors of half-hourly gap-filled estimates and a normal distribution with a mean equal to the corresponding gap-filling estimate and a standard deviation of σ_i_. From 50 Monte Carlo simulations of the observation period, with missing data replaced by an estimate randomly selected from its corresponding Gaussian distribution, 50 estimates of annual NEE and ET and their corresponding standard deviation were obtained.

Gaps in half-hourly net CO_2_ and water vapor fluxes were filled and then NEE was partitioned into GPP and RE by using the “night-time data-base estimate” method^55^. Assuming GPP = 0 at night, RE is obtained as an exponential relationship between RE and temperature following the Van´t Hoff equation^56^:

1 $$NE{E}_{night}=R{E}_{night}={R}_{eco,ref}{e}^{B(T \mbox{-} Tref)}$$

where *R*_eco,ref_ is RE at reference temperature (*T*_*ref*_, set to 283.16 K) considered as the base respiration, *B* is a site specific parameter, *T* the observed soil temperature (K),. The resulting exponential relationship is extrapolated to daytime and then GPP is obtained as the difference between RE and measured NEE. We used bin widths of 2 K and a minimum bin size of n = 10 to calculate mean values of RE and to reduce undesired effects of heteroscedasticity.

Daily and ten day estimates of NEE, GPP, RE and ETa were obtained as the sum of the half hour values over the day or ten days, respectively. Annual estimates correspond to the sum of daily values for the entire year from 2010 to 2012.

#### Environmental variables

Incident PAR and *R*_*n*_ were obtained from a quantum sensor (LI190SB; Li-Cor) and a NR-Lite sensor (Kipp and Zonen, Delft, Holland), respectively, both mounted at 4 m height. The soil heat flux was measured for both sunlit and (canopy) shaded soil using 4 plates installed at 8 cm depth (HFP01, Hukseflux, Delft, Holland), and soil temperature *T*_*s*_ by two integrated soil thermocouples (TCAV, CSI) at 2 and 6 cm depth. PAR, *R*_*n*,_ soil heat and temperature data were registered every 10 s, and half-hour averages were stored in a data logger (CR5000, CSI).

Volumetric SWC was measured daily via time domain reflectometry (TDR) (CS616, CSI) at a depth of 4 cm. TDR sensors were calibrated before starting the experiment using soil samples of the location under study with known water content. Along with these data, daily rainfall and screen air temperature data from 1994 through 2012 were obtained from a weather station located 4.3 km southeast of the study site (33°04′33′′S 70°46′07”W), and long-term rainfall data (1950–2013) from a weather station located in Santiago (33°26′42′′S 70°40′58′′W; Fig. 1b) were used to contextualize drought conditions during the experiment. Additionally, the above-mentioned data were used to compute hourly reference evapotranspiration (ET_0_) according to the FAO-56 methodology^57^.

To study the dependence of GPP and RE on SWC we calculated the relative soil water content (RWC) of the most active period (July to September) and classify the observations into the classes: Low (0 < RWC < 0.3); Medium (0.3 < RWC < 0.6) and High (RWC > 0.6). The equation used to calculate RWC was:

2 $$RWC=\frac{SWC-SW{C}_{\min }}{SW{C}_{\max }-SW{C}_{\min }}$$

where SWC_min_ (0.04) and SWC_max_ (0.49) correspond to the minimum and maximum volumetric SWC values, respectively.

#### Enhanced Vegetation Index

Vegetation indices derived from satellite imagery are commonly used to assess ecosystem biomass. In this work, the Enhanced Vegetation Index (EVI) from the National Aeronautics and Space Administration’s (NASA) Moderate Resolution Imaging Spectroradiometer (MODIS) was used as an estimate of the area-average active aboveground biomass so as to investigate its relationship with carbon fluxes and the anomalies induced by drought conditions. Sixteen-day composites at 250 m spatial resolution from 2000 to 2012 (MOD13Q1 product) were obtained for the study site by averaging EVI values for the 13 pixels (3.25 ×3.25 km) around the EC tower with the same land use. Differences in annual and monthly EVI mean values were assessed using a one-way ANOVA multiple comparison test (Fisher’s LSD; *p* < 0.05).

## Electronic supplementary material

Supplementary Information
