# Supplementary material for: Soil water content effects on net ecosystem CO2 exchange and actual evapotranspiration in a Mediterranean semiarid savanna of Central Chile
Source: Sci Rep. 2018 Jun 5;8:8570. doi: 10.1038/s41598-018-26934-z (PMC5988705; doi:10.1038/s41598-018-26934-z)
Supplement: Supplementary file 1 — Supplementary Information [file 41598_2018_26934_MOESM1_ESM.pdf]

Soil water content effects on net ecosystem CO_2 exchange and actual evapotranspiration in a Mediterranean semiarid savanna of Central Chile

Francisco J. Meza[a,b†], Carlo Montes [c], Felipe Bravo-Martínez[a], , Penélope Serrano-Ortiz[d,e], and Andrew S. Kowalski[e,f]

[a] *Facultad de Agronomía e Ingeniería Forestal, Pontificia Universidad Católica de Chile, Santiago, Chile*
[b]*Centro Interdisciplinario de Cambio Global, Pontificia Universidad Católica de Chile, Santiago, Chile*
[c] *NASA Goddard Institute for Space Studies, New York City, NY USA*

[d]*Departamento de Ecología, Universidad de Granada, Granada, Spain*
[e]*Andalusian Institute for Earth System Research (CEAMA-IISTA), Granada, Spain*
[f]*Departamento de Física Aplicada, Universidad de Granada, Granada, Spain*

Supplementary material

Table S1. Eigenvalues, explained variance, and eigenvectors associated with the 9 principal components (PC) after the application of the Principal Component Analysis. See text for acronyms.

PC	Eigenvalue	Explained variance (%)	Eigenvectors								
			GPP	RE	ETR	SWC	PAR	R_n	T_a	T_s	VPD
PC 1	5.32	59.10	-0.23	-0.27	-0.23	-0.35	0.37	0.36	0.38	0.41	0.35
PC 2	1.60	17.77	0.53	0.46	0.41	0.23	0.21	0.28	0.27	0.12	0.27
PC 3	0.94	10.39	-0.32	-0.32	0.58	0.24	0.33	0.36	-0.25	0.02	-0.31
PC 4	0.40	4.42	0.52	0.01	-0.37	-0.24	0.36	0.26	-0.33	0.05	-0.48
PC 5	0.31	3.49	-0.36	0.62	0.25	-0.57	0.02	-0.07	0.02	0.13	-0.29
PC 6	0.22	2.49	-0.38	0.43	-0.47	0.59	0.10	0.19	0.00	0.18	-0.14
PC 7	0.12	1.28	0.16	-0.18	0.09	0.09	-0.49	0.02	0.33	0.60	-0.46
PC 8	0.07	0.73	-0.04	0.07	-0.05	-0.16	-0.58	0.73	-0.21	-0.20	0.15
PC 9	0.03	0.34	-0.04	-0.05	-0.06	-0.01	0.07	0.16	0.68	-0.60	-0.37

†*Corresponding author:*F. J. Meza, Departamento de Ecosistemas y Medio Ambiente, Pontificia Universidad Católica de Chile, Av. Vicuña Mackenna 4860, Macul, Santiago, Chile.
E-mail: fmeza@uc.cl
